# Walking Behavior in Temuco, Chile: The Contribution of Built Environment and Socio-Demographic Factors

**DOI:** 10.3390/bs12050133

**Published:** 2022-05-05

**Authors:** Mohammad Paydar, Javier Arangua Calzado, Asal Kamani Fard

**Affiliations:** 1 Escuela de Arquitectura Temuco, Facultad de Ciencias Sociales y Artes, Universidad Mayor, Av. Alemania 0281, Temuco 4780000, Chile; javier.arangua@umayor.cl; 2 Universidad Católica del Maule, San Miguel 3605, Talca 3460000, Chile; asal.kamanifard@gmail.com

**Keywords:** walking behavior, socio-demographic factors, built environment, destination type

## Abstract

The increase in active travel contributes to maintaining the minimum rate of physical activity and therefore has a positive impact on inhabitants’ public health. The level of walking for daily transport has decreased significantly during the last decades in Temuco, Chile. This study examined the contribution of socio-demographic factors, active family environment, and built environment factors to walking behavior and walking level based on three types of destination in Temuco. The results of Encuesta Origin Destino (EOD 2013), geographic information system (GIS), and, finally, hierarchical multiple regression analysis were used to examine the objectives. Correlations were found between total walking behavior, walking level based on three destination types, and several socio-demographic factors such as age, gender, and access to TV and Internet. Furthermore, correlations were found between walking behavior and active family environment, as well as several built environment factors. For instance, the higher mixed land use as well as number of parks and plazas contribute towards more overall walking as well as two types of walking. Identifying that most persons who walk come from low-income families and the negative impact of network connectivity on overall walking are the major differences between this context and developed countries.

## 1. Introduction

The use of private cars for daily transport leads to more air pollution, which is one of the main problems in the cities of southern Chile. Non-motorized modes of transportation such as walking and cycling, on the other hand, are the most sustainable forms of transportation due to their accessibility, lesser emissions, and cheaper prices [1]. In addition, the increase in active travel contributes to maintaining the minimum rate of physical activity and therefore has a positive impact on inhabitants’ public health [2,3]. The need for an increase in active modes of travel for daily transport was also emphasized during the recent COVID-19 pandemic, when the use of public transport presented risks for the inhabitants’ health [4]. As of late April 2020, more than 150 communities have expanded their emergency cycling and walking infrastructures to strengthen their resiliency in the face of the COVID-19 pandemic [5].

**Figure 1:**
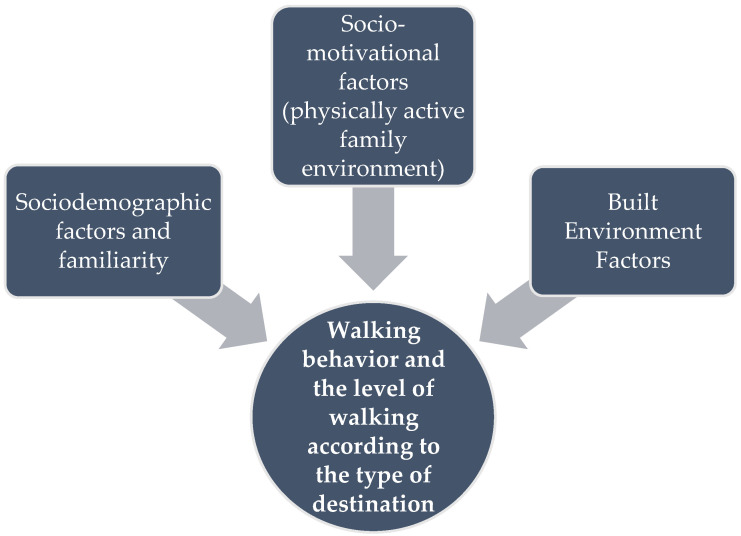
The Research Framework.

Chile is known as a semi-developed country or a country in transition towards being a developed country [6]. Temuco, the capital of the Araucania region, is one of the medium-sized southern cities with a population of almost 300 thousand people according to the 2017 Census. The rate of walking decreased (5%), while the rate of using private cars increased (7%) in daily transport trips from 2003 to 2013 in this city [7]. However, walking has still remained the most common transport mode up until today among lower-middle income groups in Chile [8]. This is confirmed by the analysis of walking trips in Temuco, which shows that 24% of the total trips in Temuco are dedicated to walking trips [7]. Thus, the rate of walking for daily transport in this city has decreased in recent decades in spite of its potential for being the main travel mode of inhabitants in this medium-sized city. In this regard, the main objective of this research is to clarify the main socio-demographic, social, and built environment factors contributing to improve walking behavior in Temuco (Figure 1). It should be noted that most of the factors in regards to walking have been found in developed countries, and there is still a lack of such studies in the countries in transition towards developed status such as Chile.

Walking behavior is governed by a complex interaction between individual and environmental (physical and social) characteristics, according to ecological models [9,10]. Previous research has shown that a variety of personal and sociodemographic, social, and built environment elements play a role in improving walking habits [11,12,13,14,15]. In addition, some prior research on walking behavior looked at the relationship between walking behavior and its contributing elements based on the specific aims of walking trips, such as walking to work, walking to school, and other sorts of walking trips [16,17,18,19,20,21,22]. These studies revealed that the influence of many elements on walking behavior changes depending on the goal of the walks. In this regard, the present study investigates the contribution of the selected factors towards walking behavior based on the different purposes of the walking trips as well (Figure 1). The following are the study’s research questions:

In this city, what socio-demographic, sociological, and built environmental elements influence walking behavior?

What effect does the goal of the walks have on the relationship between walking behavior, socio-demographic factors, social factors, and built environment factors?

What are the primary differences between developed countries and Chile’s context as an internationally relevant, semi-developed (or in transition towards developed) country in terms of the factors that influence walking behavior?

## 2. Literature Review

In terms of socio-demographic parameters, age [23,24,25], gender [23,25], holding a driver’s license [26], and educational level [23,24,27] are all consistently connected to walking. For instance, the walking rate usually decreases for older people [23]. Other research [27,28,29] discovered links between walking and having a job, income, marital status, the number of automobiles, the number of adults, and the Body Mass Index (BMI). The influence of attitudes toward walking as well as lifestyle on walking behavior has also been demonstrated by previous studies [30,31,32,33,34]. Previous research on walking behavior has also shown a link between familiarity with the walking environment and walking behavior [11,35]. Furthermore, the social environment refers to the impact that friends and family might have on a person’s walking habits [36]. Most of the social aspects associated to walking were first discovered in the literature on physical exercise and subsequently applied to walking [23]. A positive social environment has regularly been shown to improve physical activity and walking in previous studies [37,38]. The existence of a role model has been found to be a motivating factor for walking and physical activity among the social factors related with walking [23,39]. When it comes to walking, role models are persons who walk and urge others to do so as well. Physical exercise and walking are directly related with the support of family and friends, such as a partner or friends who are physically active [39,40]. A physically active family setting has a significant influence in encouraging people to engage in more physical exercise and walking, according to the role model indicators [41]. Some research, however, found that this component had no effect on walking or overall physical activity [23].

In terms of the built environment, most of the research found that measures of land use, such as population/residential density, housing density, and mixed land use, have a beneficial impact on walking behavior [14,32,42,43,44,45]. Likewise, access to a variety of destinations such as shops, services, and work is one of the most important built environment attributes which contributes to walking behavior [15,32,34,35,45,46,47]. The pedestrian environment, which includes variables such as ease of street crossing, sidewalk presence, sidewalk breadth, sidewalk continuity, well-connected street network, street density, and terrain, has a favorable impact on walking behavior [32,35,42,43,48]. Walking facilities, such as sidewalk condition and benches, were also mentioned as factors that influence walking habits [15,35,49]. Walking for transportation has also been linked to traffic safety and personal security [35,42,47,49,50,51]. Furthermore, characteristics such as visual interest, visibility of landmarks along pathways, vistas of public gardens, visible activity, street trees, and cleanliness all influenced walking behavior [3,35,42,52].

Finally, some past research on walking behavior focused on walking trips for specific goals, such as walking to work or to school. Craig et al. [16], for example, investigated the relationship between walking to work and community design elements. Plaut [17] looked studied the link between non-motorized commuting, such as walking and cycling, and occupational and socioeconomic characteristics. Other research looked at the relationship between university students’ walking behavior and personal and built-environmental characteristics [21,53].

## 3. Materials and Methods

Temuco, the capital of the Araucania region, is one of the medium-sized southern cities with a population of about three hundred thousand people according to the 2017 Census. The information of Travel Diary Data taken from Origin Destination Survey (Encuesta Origin Destino; EOD Hogar y Viajes), conducted by the ministry of transport, Chile, Temuco 2013, is used to measure walking behavior and the relevant socio-demographic factors [7]. Previous research has employed a travel diary survey to assess walking behavior as well as the socio-demographic characteristics that influence it [54]. EOD is a general purpose survey covering a wide range of issues including transport and travel. On the day of travel registration, participants filled out a travel diary that described all their journeys. The origin, destination, purpose (assigned to all stages that made a given journey), distance, and mode of transport were all collected for each journey stage. Because walking was a characteristic assigned to each journey stage, each participant’s walking behavior could be measured if walking was included in his or her travel diary (minutes of walking dedicated to each walking trip). EOD 2013 was used to choose a sample of 1721 people who walk in Temuco on a regular basis. In fact, this sample includes the total number of the respondents whose walking was recorded as part of their daily travel modes in EOD 2013. The number of respondents was adequate for our analysis when compared to previous studies, which were implemented in a similar context, in terms of size and population [55].

Household size, employees per household, adults per household, income (family or individual), number of vehicles in each household, possession of a driver’s license, gender, employment status, and age were all provided by EOD Home and Travel. Because most of the respondents fell into these two categories, a continuous variable such as income has been turned into a categorical variable with two categories of low versus middle income. In addition, duration of living has been used as the indicator for Familiarity. It was measured through two categories of less than one year versus more than one year of living in the house, which could be better representative of a low versus medium level of familiarity with the walking environment. Finally, education was divided into three categories of low educational level, intermediate educational level, and high educational level.

The selected indicators, namely, physically active family environment, as well as the role model as a factor, were measured objectively using two variables of “number of walking trips” and “proportion of walking trips compared to total trips in each household”. Because walking is a sort of physical activity, these measures reveal each family’s tendency to be active. This could inspire members in each home to walk more. In addition, the attributes of neighborhoods which pertained mainly to land-use and built environment variables were obtained from the Cadaster Department of the municipality of Temuco.

The built environment factors included density, mixed land use, and destination accessibility, connectivity, traffic safety, personal security, aesthetics, type of houses, and topography (slope). Density was measured through both population density (number of inhabitants in each zone) and housing density (number of housing units in each buffer). Entropy index was used to measure land-use diversity, which includes five main types of land use in this city: residential, commercial, services, and educational and health centers, as well as hospitals. This study also measures the population–employment entropy index as other indices of mixed land use which calculates the job–house balance in a given area. The population–employment entropy criteria identify a value between zero to one which could be representative of residential-dominant or commercial-dominant land use for each neighborhood. The numbers of each type of these land uses in each buffer were also measured to calculate destination accessibility. Connectivity was measured using three indicators of Link Node Ratio (Links per unit of area (streets)/# Nodes per unit of area), Intersection Density (Real nodes area/Area), and Street density (Total street length per unit of area/area) [56]. For instance, a higher intersection density reveals greater network connectivity and, consequently, higher permeability in neighborhood buffers. The literature suggests that a link–node ratio of 1.4 or 1.2 indicates connected networks. Traffic safety was measured using reported pedestrian accidents during the last year, and personal security was measured using the number of reported crimes during the last year taken from the city police station. Furthermore, aesthetics was measured through the calculation of two indicators including the number of trees and number of parks and plazas in each buffer zone. Finally, the type of housing, whether villa or apartment, was extracted from the EOD survey, and the slope was measured using three values: “high slope”, where most streets of the buffer have more than a 15% slope; “medium slope”, where most streets of the buffer have between a 5% to 15% slope; and “low slope”, which is less than a 5% slope.

The built environment variables were measured objectively using Geographic Information Systems (GIS) tools in the buffer zones with a radius of 400 m around the household in EOD which undertook the walking trips. The buffer size is a generally accepted “walkable” distance in existing research and could capture attributes of built environments immediate to one’s residence [57]. Some information in relation to population density was also taken from CENSO 2017, Temuco, Chile. The data were analyzed using SPSS software version 23.0. Hierarchical multiple regression analysis was used to predict a dependent variable from the independent variables. Several models have been made in both total walking and walking based on destination types to reach the best-fitting models. Firstly, models that contained key control variables related to the socio-demographic attributes of respondents and their households were used in a way that each new variable enhances the explanatory power of the model. In the next step, the social factors were added. After that, we examined whether neighborhood-scale built environment variables added significant statistical explanatory power to person and household-level control variables, as well as social factors. In each step, the variables which showed high multi-collinearity (VIF > 5) were deleted, and those which enhanced the explanatory power of the models were retained until reaching the best-fitting models. Therefore, the models that were chosen and that are presented in this article represent the combinations of control variables and representations of the built environment factors that were interpretable, minimally inter-correlated, and consistent with the theory

## 4. Results

### 4.1. Descriptive Statistics

Table 1 shows the descriptive statistics for the socio-demographic variables and familiarity. Most of the respondents are female (59.1%) as compared to male (40.9%). In addition, most of the respondents do not work or are retired (72%). Most people who have income are low-income, with 63.8 percent receiving less than 324 thousand Chilean pesos each month. The majority of respondents (91.6%) live in houses, compared to 8.4% who live in apartments, and the majority of them are the owners of their homes and apartments (75 percent). Added to this, most respondents do not have a driver’s license (84.5%) as well as the vehicles in each household (66.6%). Furthermore, the majority of respondents have both primary and secondary school education (92%). Finally, each household has an average of 4.12 family members, and most respondents are very familiar with the walking environment because they have lived in their current home for more than a year (93.6 percent). Table 1 also shows the mean value of the variables regarding social factors and built environment variables.

Walking to educational destinations, walking to workplaces, and walking for shopping are the three types of walking trips that account for the biggest percentages of walking trips (Table 2). As a result, these three types of walking have been chosen to investigate the role of socio-demographic, social, and built-environmental elements in walking behavior dependent on the aim of the walks.

### 4.2. The Factors Influencing Walking Behavior (Overall Walking)

Table 3 shows the results of the best-fitting model regarding the regression analysis between walking behavior and its contributing factors. R2 (0.102) shows that the independent variables of this study exhibit an explicative power of almost 0.102 in predicting the dependent variable (walking behavior). This rate of R2, for the most part, does not indicate a high total contribution for the independent variables regarding walking behavior. However, considerable correlations were identified between some of the independent variables and walking behavior.

The most significant positive link with walking behavior was age (β = 0.159, *p* = 0.000), indicating that older adults walk much more in this city. Men walk much more than women (β = 0.106, *p* = 0.000), according to the findings. The number of people in the family influences the amount of walking (β = 0.075, *p* = 0.025). In contrast, a larger number of daily trips in the home leads to a reduction in walking, and vice versa (β = −0.132, *p* = 0.000). Respondents who work and earn a monthly income walk much more than those who do not (β = 0.081, *p* = 0.002). Additionally, people who do not have a driver’s license walk much more than those who have (β = −0.047, *p* = 0.079). The “Number of walking trips in each family” had a significant positive connection with walking habits (β = 0.135, *p* = 0.000) among the social components. This link demonstrates that a larger number of walking trips in the home contributes to increased walking activity.

From the built environment factors, housing density showed the significant positive correlation with walking behavior (β = 0.090, *p* = 0.003), indicating that increases/decreases in housing density are related to increases/decreases the level of walking in this city. A higher number of parks and plazas contribute to improving walking behavior (β = 0.089, *p* = 0.000). In addition, a higher Link node ratio as one of the indicators of network connectivity contributes to less walking and vice versa (β = −0.010, *p* = 0.003). Similarly, a higher number of educational destinations—leading to greater accessibility to this type of destination—contributes to a lesser walking level and vice versa (β = −0.073, *p* = 0.004). Finally, mixed land use showed a significant positive correlation with walking level (β = 0.087, *p* = 0.002), which shows that higher diversity of land uses contributes to improving the level of walking.

### 4.3. The Factors Influencing Walking Behavior Based on Three Types of Destination

Table 4 shows the results of the best-fitting model regarding the regression analysis between the level of walking to/from workplaces and its contributing factors. According to R2 (0.232), the independent variables exhibit an explicative power of almost 0.232 to predict the level of walking to/from destinations.

Age was shown to have a significant positive correlation with walking to to/from workplaces, which suggests that older people walk significantly more to/from workplaces (β = 0.171, *p* = 0.005). Men walk significantly more than women to their workplace (β = 0.102, *p* = 0.078). Greater access to TV in each household has the negative impact on walking (β = −0.102, *p* = 0.082). From the social factors, “proportion of walking trips to total trips in household” showed a significant positive correlation with walking to the workplace (β = 0.175, *p* = 0.006).

From the built environment factors, the highest significant correlation is found between total accident rates as the main indicator of traffic safety and the level of walking to/from workplaces (β = −0.176, *p* = 0.043). This correlation shows that higher accident rate contributes to less level of walking to/from workplaces and vice versa. The crime rate has a significant correlation with walking behavior, where a higher crime rate contributes to less walking to/from workplaces and vice versa (β = −0.159, *p* = 0.055). People walk to their workplace considerably more frequently in areas with a higher number of parks and plazas (β = 0.152, *p* = 0.017). Housing density showed the significant positive correlation with walking to/from workplaces (β = 0.131, *p* = 0.024). Furthermore, mixed land use was found to have a strong beneficial relationship with walking levels (β = 0.130, *p* = 0.062). Finally, accessibility to educational destinations has a negative impact on walking to the workplace (β = −0.111, *p* = 0.061).

In regards to walking to educational destinations (R2: 0.157) (Table 5), men walk significantly more than women (β = 0.080, *p* = 0.061). Age was shown to have a significant positive correlation with walking to educational destinations, which indicates that older students/people walk more to reach this type of destination (β = 0.171, *p* = 0.005). The respondents with a higher level of education walk significantly more to reach the educational destinations (β = −0.172, *p* = 0.014). Those without a driver’s license walk substantially longer distances to educational destinations than those who do, and vice versa (β = −0.146, *p* = 0.002). Furthermore, the home owners, walk significantly less to the educational destinations and vice versa (β = −0.100, *p* = 0.041). In regards to social factors, a greater “proportion of walking trips to total trips in household” contributes to more walking in order to reach this type of destination (β = 0.101, *p* = 0.046). In addition, regarding the factors of built environment, a higher number of educational destinations—leading to greater accessibility to this type of destination—contributes to more walking to educational destinations (β = 0.181, *p* = 0.001).

Regarding walking for shopping (Table 6), according to R2 (0.231), the independent variables exhibit an explicative power of almost 0.231 to predict the level of walking for shopping. Among the people who work, those who work at home walk more than others for shopping (β = 0.267, *p* = 0.000). People with low monthly income walk significantly more for shopping and vice versa (β = 0.186, *p* = 0.006). Greater access to the Internet leads to a significant reduction in walking for shopping (β = −0.143, *p* = 0.024). The “Number of persons in each household” was shown to have a significant negative correlation with walking for shopping (β = 0.124, *p* = 0.060). Among the social factors, a greater “proportion of walking trips to total trips in household” contributes to more walking for shopping (β = 0.193, *p* = 0.002).

Mixed land use had the strongest significant positive connection with walking for shopping among the built environment characteristics (β = 0.245, *p* = 0.010), which shows that a higher diversity of land uses contributes to improving this type of walking. People walk for shopping considerably more frequently in areas with a higher number of parks and plazas (β = 0.134, *p* = 0.036). Finally, areas with steeper slopes lead to a reduction in this type of walking (β = −0.166, *p* = 0.026).

**Figure 2:**
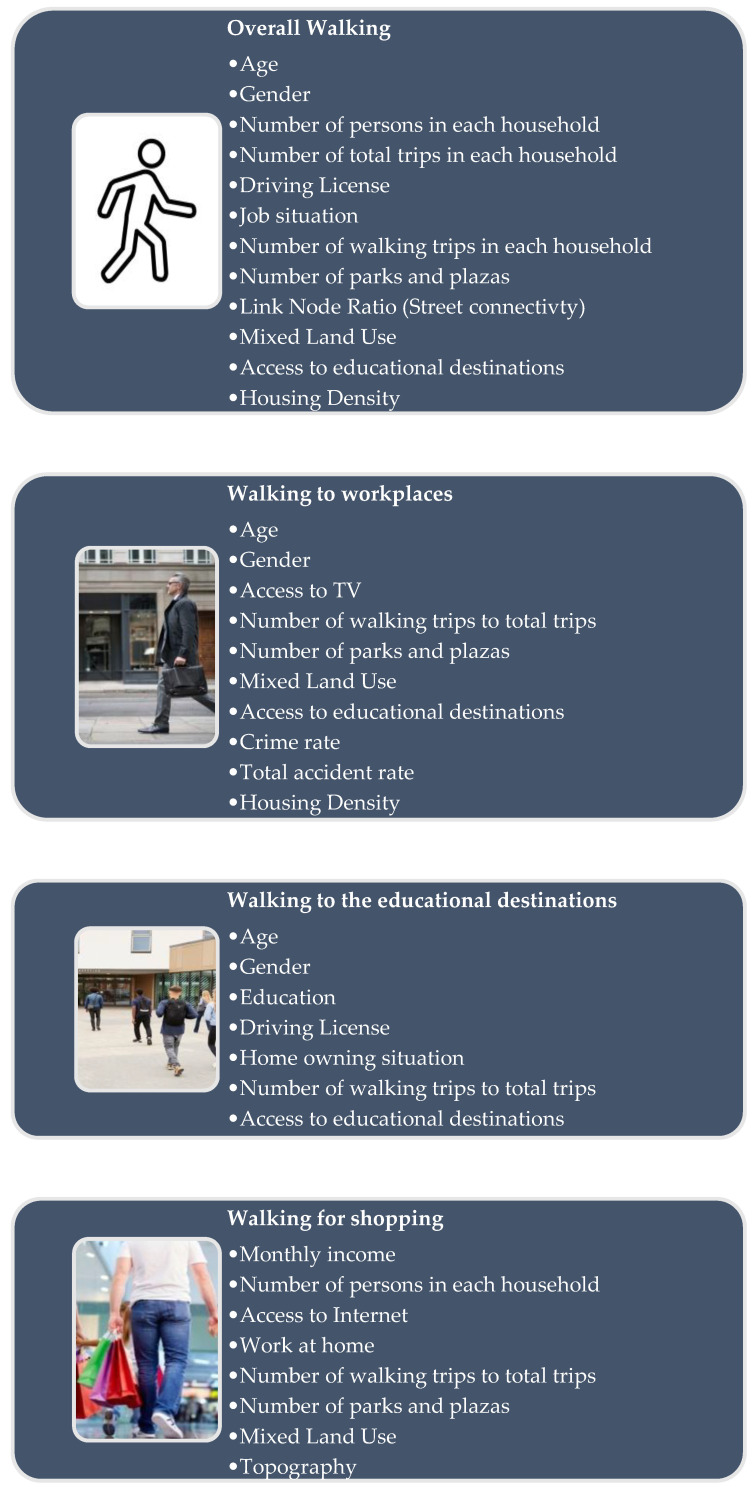
The summary of the correlated factors to overall walking and the three types of walking, including walking to workplaces, walking to reach the educational destinations, and walking for shopping (Pictures taken from https://www.vecteezy.com; https://www.drweil.com; https://www.thecompleteuniversityguide.co.uk; https://www.justlovewalking.com/. All the mentioned websites were accessed on 30 April 2022).

Figure 2 depicts a summary of the socio-demographic, societal, and built-environmental elements that influence overall walking, as well as the three categories of walking: walking to work, walking to educational destinations, and walking for shopping.

## 5. Discussion

### 5.1. The Influence of Socio-Demographic and Social Factors on Walking Behavior

According to descriptive analysis, most of the respondents who had an income came from low-income families (63.8 percent). As a result, many of the people who walk in Temuco come from low-income households. This is consistent with the findings of Herrmann-Lunecke et al. [8], who discovered that walking is the most popular mode of transportation in Chile, particularly among low-income groups. Furthermore, people with low monthly income walk significantly more for shopping than people with average/high monthly income. This result is in line with previous studies which found that greater monthly income contributes to sedentary behavior, as well as less walking and vice versa [58,59]. One interpretation in this regard is that those with lower incomes may walk longer distances to the shops that have the goods they need at cheaper prices. Because one product could have different prices in different shops at varying distances from home in Chile. On the other hand, those who have a higher monthly income may use the nearest shop to their home regardless of the price difference in different shops; in this way, their daily walking for shopping is reduced. However, an analysis of the distribution of land uses and shops in different neighborhoods is also required to prove this theory, and future studies could investigate this relationship in more depth.

Furthermore, overall walking improves among persons who have a job and a monthly income. These findings should be seen in the perspective of low-to-middle-income families, which account for most persons who walk in this city. Those who work and earn a monthly income are responsible for their families and must be more active to meet the demands of other family members; as a result, they walk more than their other family members. Moreover, among the people who work, those who work at home walk significantly more than others for shopping. A low percentage of respondents (5.2%) work at home and according to our experience in this city most of these jobs are service work such as groceries and special workshops. One interpretation could therefore be that these people need to buy various necessities in regards to their home-based businesses, and as a result, their walking rate increases.

It was found that most of the people who walk in this city do not have a private car and/or a driver’s license. Thus, private cars generally have no role in the lives of people who walk in this city. Furthermore, for people who do not have a driver’s license, the overall walking and walking to educational places improves. Previous research backs up this conclusion, showing that the number of automobiles in a household and holding a driver’s license reduces active travel and walking [36,60]. Furthermore, these findings reveal an incompatibility between walking and private car use for daily transportation of persons who walk in this city, particularly those who walk to educational destinations. In terms of walking to educational destinations among younger people and millennials in this city, the majority of those who walk to educational destinations are younger people and millennials born after 1980/1990. Previous studies have shown that unlike developed countries, where millennials rely more on active travel in their daily trips, young people and millennials in developing countries have a greater tendency to use private cars for their daily trips to reach the universities [61]. There could be a similarity between this context and developing countries rather than developed ones in terms of the tendency of millennial for less motivation to walk in their daily trips. However, the current study did not look at other forms of transportation, such as private vehicles, and this preliminary conclusion would need to be investigated further in future studies.

Moreover, older people are considerably more likely to walk in this city. When it comes to different types of walking journeys based on the destination, older people walk more to work and to school. According to our findings, the bulk of persons who walk to reach educational locations are adolescents and young people (10 to 29 years old; 89 percent), also known as millennials, who were born between 1980 and 1990. Further analysis also showed a significant correlation of age with walking behavior, which is due to the significant differences between age groups under 60 years old in relation to walking behavior. Moreover, the three elderly age groups between 60 and 100 years old did not show a significant correlation with walking behavior. This contrasts with prior research, which revealed that physical activity and walking behavior decreases with age [23,62,63]. One interpretation in regards to walking to educational destinations is that the adolescents—in contrast to young adults—are not able to ward off the potential health risks during walking. As a result, they may be more reliant on other modes of transportation, such as school service, rather than walking independently from their home. In addition, men walk significantly more when compared to women. Similarly, men walk much more than women to get to work and educational institutions. Gender has an impact on walking behavior, according to previous studies [24,64]. One interpretation is that this reduction in walking rate is due to the influence of environmental factors such as perceived insecurity and the potential threats women may encounter when walking, since previous studies have shown the impact of perceived insecurity on reducing women’s walking in Chile [51]. In this regard, women are generally more vulnerable than men regarding potential threats that may occur during their walking [65,66]. Future research could investigate the other obstacles that women face when walking in this city.

Furthermore, persons who have televisions in their houses walk substantially less to and from work than those who do not. This is an intriguing finding that demonstrates how technology devices have a negative impact on physical activity and walking. Previous research has showed that watching more television leads to greater sedentary behavior and less physical activity, such as walking [67,68]. However, walking to workplaces is considered to be a mandatory activity which does not have the flexibility to be adopted at any time and place. This suggests that there could be additional reasons outside the positive influence of television on sedentary behavior, which future research should look at. Similarly, more access to the Internet contributes to less walking for shopping and vice versa. Information and communication technologies (ICT) have had a major impact on travel behavior patterns within the last decades [69]. Along with this global trend, the rate of online shopping through different related mobile apps has increased in Chile within the last decades [70], and this could be the reason for finding a reduction in walking for shopping where there is greater Internet access. This result is also in line with the findings of the previous studies, which showed the impacts of technology on enhancing sedentary behavior which may finally contribute to decreasing the level of physical activity and walking [71,72]. Future studies may investigate the role of access to Internet and the ways in which it contributes to the level of physical activity and walking in this city.

Furthermore, people with a higher educational level walk more toward educational destinations. This is in line with the results of previous studies, which found that people with a higher educational level walk more than others [23,24,27]. However, the reason for this finding and the difference in walking frequency between adolescent and university students could be more closely related to other aspects such as less independence of adolescents compared to university students rather than related to their educational level.

Moreover, from two indicators of “a physically active family environment” used in this study, a greater “number of walking trips in each household” contributed to enhance the overall walking and the higher “proportion of walking trips to total trips in household” contributes to an increase in two types of walking, including walking to workplaces and walking to educational destinations. People are more likely to walk if the proportion of walking trips in their family’s overall trips is increased. In addition, the “number of persons walking in each household” adds to increased retail walking. This is consistent with prior research, which found that role models and an active family environment have a considerable impact on increasing physical exercise and walking [39,40,41]. The policy makers of this city should pay attention to this motivational factor in order to improve walking behavior in this city. This association suggests that there may be more social factors influencing walking habit in this city. Future research could go deeper into this topic.

### 5.2. The Influence of Built Environment Factors on Walking Behavior

Regarding the built environment factors, the number of parks and plazas showed a significant positive correlation with total walking and two types of walking including walking for shopping and walking to workplaces. People in those areas which presented the highest numbers of parks and plazas, walk significantly more to all types of destination, including workplaces and shops. This finding is supported by previous studies, which showed the importance of aesthetic factors such as the presence of recreational green spaces to encourage walking [35,42]. However, these studies showed the contribution of recreational green spaces to walking for recreation rather than transport walking [73]. Thus, a direct association between the number of parks and plazas and walking to workplaces is not well supported by previous studies. One interpretation in this regard is that many workplaces are located around the park and plazas in this city and therefore increasing the number of parks and plazas contributes to a greater number of workplaces; therefore, walking to workplaces is increased. In addition, this city does not have a high number of recreational spaces such as parks and plazas in the different city sectors, especially in peripheral areas, and several of the current recreational spaces are not in good physical condition [7]. This finding is therefore applicable for the city’s urban and transport policy makers; they should raise the quality and quantity of recreational and green spaces such as parks and plazas in different sectors in order to improve walking in this city.

Mixed land use is the other built environment factor which contributes to enhancing overall walking and walking for shopping as well as walking to workplaces. This finding is supported by previous studies which found a positive correlation between mixed land use and walking behavior [32,42]. It is also to be noted that the mixed land use is higher in the city center and its surrounding areas, where the land uses other than residential land use, including commercial, service, health, and educational land uses, increase significantly. Indeed, enhancing mixed land uses in the city center and its surrounding areas has been part of the compact city approach, which is observed in these areas within the last decades in Temuco. One of the most well-known methods to sustainable urban development is the compact city [74], which leads to an increase in sustainable transportation modes such as walking and cycling [75]. This finding supports the positive impact of the compact city approach, initiated and strengthened in Temuco in recent decades on enhancing the walking level and sustainable urban transportation in this city. Therefore, it is to be strengthened by urban/transport policy makers of this city at future.

Furthermore, the Link Node Ratio, as one of the indicators of connectivity, was shown to have significant negative correlations with overall walking level. This shows that a better-connected street network contributes to a decrease in the level of walking. In other words, people walk more when there is a more disconnected urban street pattern. In contrast to prior research, which concluded that a better-connected street network improves walking behavior, this is not the case [76,77]. One interpretation in regards to these findings is that such results could be considered in the light of the crime level and sense of insecurity as a factor, for which correlation to walking to workplaces has also been found. The number of gated communities within residential neighborhoods has increased within the last decade in different cities of this country in order to enhance actual security as well as the sense of security among the residents. Gated communities are usually residential areas restricted by fences and walls. In fact, the actual crime rate as well as fear of crime create situations where the inhabitants prefer to walk more in disconnected street patterns than connected ones. This situation regarding the design and layout of streets in relation to walking behavior could also describe one of the major differences between this context and the developed countries in terms of the association between the factors of built environment and walking behavior.

Moreover, a higher housing density is associated with the increase in overall walking level as well as two types of walking, including walking to workplaces and walking for shopping. The positive effect of residential/population density on increased walking has been indicated by previous studies [15,78,79]. In fact, the increase in residential/population density—together with mixed land uses—contributes to reducing travel time and distance between origins and destinations and promoting walking as a means of transportation [56]. This result shows that the increase in the level of walking can constitute a supporting argument for the urban densification policy in Temuco. More precisely, this result supports the positive impact of the compact city approach in improving walking and sustainable transportation that has been initiated and reinforced especially in city center and its surrounding areas in recent decades.

Lower accident rates, as an indicator of traffic safety, contributes to enhancing walking to workplaces. This finding is supported by the previous studies which found that more traffic safety contributes to enhance walking [35,80]. In addition, a higher crime rate is the other built environment factor which contributes to a decrease in walking to workplaces. Chile is considered to be a country with a moderate to low crime rate, when compared to the average amount of crime around the world [81]. However, the different types of crime in the Araucania region are mostly concentrated in Temuco [82]. In addition, previous studies have shown the effect of perceived insecurity in reducing the level of walking in residential areas of Santiago, Chile [51]. This study used the real crime rate rather than perceived insecurity. The relationship between the fear of crime and the rate of crime is not well supported [83]. Indeed, the perception of insecurity does not necessarily correspond with actual insecurity [84]. However, this finding shows that there may be a strong relationship between the actual crime rate and perceived insecurity in this context, which contributes to a decrease in the level of walking. This could be further investigated by future studies in this area. The policy makers of this city could apply this finding by aiming to reduce the crime rate and its contributing social and environmental factors in order to encourage walking in this city.

Finally, topography, in terms of a steeper incline, contributes to a significant decrease in walking for shopping. The association between a steeper incline and walking behavior has been demonstrated by previous studies [85]. Some parts of this city have been built on hilly areas, and this finding shows that people walk less for shopping in these urban sectors. This finding is especially important to avoid placing the future locations of shops and malls in these hilly areas in order to encourage people walking around these commercial destinations.

## 6. Conclusions

While the use of private cars as a mode of daily transportation has increased in Temuco in recent decades, the rate of walking has decreased. This is despite the fact that walking, as the most environmentally friendly means of transportation, is better suited to the post-pandemic era, particularly in this type of medium-sized city. It was discovered that most persons who walk in Temuco are from low-income families, with a few exceptions belonging to middle-income families. This finding, which is supported by previous studies in a similar context, is the first major difference between developing countries and the developed countries in terms of walking behavior. The impact of several socio-demographic factors on walking behavior was found in this study, including age, gender, having a job, educational level, driving license, and access to TV and Internet. One of the interesting results is that more access to the Internet contributes to less walking for shopping. In addition, the contribution of different socio-demographic, social, and built environment factors to total walking, as well as walking based on three selected types of destination, were found. For instance, in regards to the factor of built environment, a greater number of parks and plazas contribute to enhancing overall walking and two types of walking including walking to workplaces and walking for shopping. And higher traffic safety as well as personal security contributes to improve the walking to workplaces. These findings were discussed and their implications were presented.

One of the most important implications of this research is the association between mixed land use as well as residential density and overall walking as well as two types of walking including walking for shopping and walking to workplaces. The approach of compact city has been started and strengthened in Temuco especially in the city center and its surrounding areas within the last decades. Thus, these areas have the higher densification and mixed land use than other urban sectors in this city. These findings support the usefulness of this approach to enhance walking and sustainable urban transport in Temuco. Therefore, these findings are applicable for urban/transport policy makers of Temuco to enhance walking, physical activity and sustainable urban transport in this city.

In addition, greater network connectivity contributes to less overall walking. This result is in contrast to the findings of similar studies in developed countries which point to the positive impact of network connectivity on walking. This finding, which highlights a major difference when compared to developed countries, could be interpreted in light of the crime rate and level of perceived insecurity being the factor in correlation to one type of walking (walking to workplaces). It can be inferred that people prefer gated communities and disconnected areas in this context of increasing their actual security as well as their perceived security.

Finally, most people who walk in this city do not have driver’s licenses or private automobiles in their households, implying that private cars are not employed as a mode of transportation for these people. Furthermore, for people who do not have a driver’s license, overall walking and walking to educational places improves. On this basis, it can be inferred that there is an incompatibility between using private cars and walking for daily trips, especially regarding millennials (school and university students), since they may tend to use private cars as well. This could be another major difference between this context and developed countries, concerning the factors that contribute to walking behavior. However, it is not conclusive and needs further consideration by future research.

This study used objective measurements of the built environment factors. The addition of more subjective or perceptual aspects of the built environment in regards to walking behavior may lead to a better understanding of the correlation between built environment and walking behavior in this city.

## Figures and Tables

**Table 1:** Descriptive statistics of socio-demographic variables, social, and built environment factors (N = 1721).

Variables	Variable Description	Frequency	Percentage	Mean
Level of walking (Min)				14.37
*Socio-demographic variables and Familiarity*				
Age (Continuous)				41.39
Gender	0 = Female	1014	59.1	0.44
	1 = Male	701	40.9	
Monthly income (Chilean peso)	0 = More than 324 thousand (Average or more)	318	36.2	0.63
	1 = Less than 324 thousand (low)	560	63.8	
Home owning situation	0 = Rented	430	25	0.74
	1 = Owner	1287	75	
Education	Primary school and lower	721	42	
	High school and similar	857	50	
	University degree	137	8	
Employment situation	0 = Without job or retired	1234	72	0.28
	1 = With job	481	28	
Access to Internet	0 = Without Internet	796	46.4	1.54
	1 = Have internet	921	53.6	
Access to TV	0 = No TV	768	44.7	1.55
	1 = Have TV	949	55.3	
Work at home	0 = No	1659	96.7	0.03
	1 = Yes	56	3.3	
Driver’s license	0 = Do not Have	1442	84.5	0.16
	1 = Have	265	15.5	
Time Living Years (Familiarity)	0 = Less than one year	111	6.4	0.94
	1 = More than one year	1610	93.6	
Number of Vehicles in each household	0 = Do not have 1 = Have	1146 575	66.6 33.4	0.33
Number of Bicycles in each household				1.07
Number of People in each household				4.12
Number of total trips in each household				11.95
*Social Factors*				
*Number of total walking trips in each household*				2.20
*Number of walking trips to total trips*				0.22
*Built Environment Variables*				Mean in 800 m buffers (400 m Radius)
Current Housing Type	0 = Apartment	144	8.4	0.92
	1 = Villa	1579	91.6	
Diversity or Mixed land	Entropy index (5 types of land uses)			0.57
	Population-employment entropy			0.30
Connectivity	Intersection density			151.91
	Link node ratio (LNR)			1.49
	Street density			19.41
Density	Population density (Number of inhabitants per buffer)			327.71
	Housing density (Number of housing units per buffer)			126.83
Accessibility	Number of Educational destinations per buffer			1.2
	Number of Health centers and hospitals per buffer			2.3
	Numbers of commercial land uses per buffer			5.9
	Number of services including bank and other types			6.1
Traffic safety	Number of reported accidents in each buffer in the last year			1.62
Personal Security	Number of reported total crime types during last year in each buffer			17.3
Aesthetic	Number of trees per buffer			82.3
	Number of parks and plazas in each buffer			0.64
Topography (Slope)	1 = High slope (more than 15%); 2 = Medium slope (between 5% to 15%); 3 = Low slope (less than 5%)			2.60

**Table 2:** Frequency of walking trips according to each type of destination in Temuco (N = 1721).

Walking Trips Based on the Purpose of the Trips	Frequency	Percentage
Toward educational destinations	509	29.6
Toward workplaces	317	18.4
Toward shopping	293	17
To meet/see someone	218	12.7
To health centers	110	6.4
For recreation	85	4.9
Other case	189	11

**Table 3:** The results of hierarchical multiple regression analysis for predicting walking behavior (overall walking) (N = 1721).

Variables	Standardized Coefficients	t	*p* Value
*Sociodemographic variables and familiarity (Level 1)*			
Gender	0.106	4.406	0.000 **
Age	0.159	5.847	0.000 **
Number of persons in each household	0.075	2.238	0.025 **
Number of total trips in each household	−0.132	−4.131	0.000 **
Familiarity	−0.033	−1.365	0.172
Driving License	−0.047	−1.755	0.079 *
Access to Internet	−0.018	−0.664	0.507
Access to TV	−0.039	−1.454	0.146
Vehicles in each household	−0.036	−1.388	0.165
Work at home	0.030	1.221	0.222
Job situation	0.081	3.170	0.002 **
*Variables of the social environment (Level 2)*			
Number of walking trips in each household	0.135	4.455	0.000 **
*Variables of the built environment (Level 3)*			
Housing type	−0.012	−0.429	0.668
Number of parks and plazas	0.089	3.565	0.000 **
Link Node Ratio	−0.10	−3.025	0.003 **
Street Density	0.036	1.180	0.238
Mixed Land Use	0.087	3.098	0.002 **
Access to educational destinations	−0.073	−2.874	0.004 **
Topography	−0.034	−1.265	0.206
Population Density	−0.026	−0.820	0.412
Housing Density	0.090	2.982	0.003 **

* *p* < 0.05; ** *p* < 0.01. Dependent variable: Walking Behavior; R Square: 0.102

**Table 4:** The results of hierarchical multiple regression analysis for predicting walking to workplaces (N = 317).

Variables	Standardized Coefficients	t	*p* Value
*Sociodemographic variables and familiarity (Level 1)*			
Gender	0.102	1.768	0.078 *
Age	0.171	2.807	0.005 **
Number of bicycles in each household	−0.093	−1.548	0.123
Number of persons in each household	0.082	0.935	0.350
Number of total trips in each household	0.099	1.185	0.237
Familiarity	−0.086	−1.555	0.121
Education (“University Degree” is Reference Category)			
Primary school and Lower degree	−0.118	−1.417	0.158
High school and similar	−0.053	0.700	0.484
Driving License	−0.078	−1.258	0.200
Access to Internet	0.061	0.981	0.327
Access to TV	−0.102	−1.746	0.082 *
Work at home	0.043	0.787	0.432
*Variables of the social environment (Level 2)*			
Number of walking trips to total trips	0.175	2.768	0.006 **
*Variables of the built environment (Level 3)*			
Housing type	0.007	0.105	0.917
Number of parks and plazas	0.152	2.407	0.017 **
Number of trees per zone	0.017	0.231	0.818
Link Node Ratio	0.019	0.227	0.821
Intersection Density	0.164	2.386	0.118
Mixed Land Use	0.130	1.877	0.062 *
Access to educational destinations	−0.111	−1.884	0.061 *
Access to commercial destinations	0.020	0.194	0.846
Crime rate	−0.159	−1.928	0.055 *
Total accident rate	−0.176	−2.030	0.043 *
Housing Density	0.131	2.271	0.024 **

* *p* < 0.05; ** *p* < 0.01. Dependent variable: Walking Behavior; R Square: 0.232.

**Table 5:** The results of hierarchical multiple regression analysis for predicting walking to the educational destinations (N = 509).

Variables	Standardized Coefficients	t	*p* Value
*Sociodemographic variables and familiarity (Level 1)*			
Gender	0.080	1.875	0.061 *
Age	0.167	2.270	0.024 **
Number of bicycles in each household	0.053	1.160	0.247
Number of persons in each household	0.049	0.945	0.345
Number of total trips in each household	−0.002	−0.033	0.974
Familiarity	−0.021	−0.434	0.665
Education (Primary school and Lower degree) (“University Degree” is Reference Category)	−0.172	−2.478	0.014 **
Driving License	−0.146	−3.099	0.002 **
Access to Internet	−0.024	−0.498	0.619
Number of vehicles in each household	−0.061	−1.321	0.187
Job situation	0.053	1.219	0.223
Home owning situation	−0.100	−2.047	0.041 **
*Variables of the social environment (Level 2)*			
Number of walking trips to total trips	0.101	1.997	0.046 *
*Variables of the built environment (Level 3)*			
Housing type	0.026	0.503	0.615
Number of parks and plazas	0.046	1.027	0.305
Number of trees per zone	−0.041	−0.760	0.448
Link Node Ratio	−0.080	−1.407	0.160
Mixed Land Use	−0.030	−0.573	0.567
Access to educational destinations	0.181	−3.502	0.001 **
Topography	−0.058	−1.287	0.202

* *p* < 0.05; ** *p* < 0.01. Dependent variable: Walking Behavior; R Square: 0.157.

**Table 6:** The results of hierarchical multiple regression analysis for predicting walking for shopping (N = 293).

Variables	Standardized Coefficients	t	*p* Value
*Sociodemographic variables and familiarity (Level 1)*			
Gender	0.041	0.697	0.486
Age	0.065	0.969	0.334
Monthly income	0.186	2.792	0.006 **
Job situation	−0.058	−0.867	0.387
Number of persons in each household	0.124	1.892	0.060 *
Education (Primary school and Lower degree) (“University Degree” is Reference Category)	0.011	0.181	0.857
Driving License	0.042	0.664	0.507
Access to Internet	−0.143	−2.274	0.024 **
Work at home	0.267	4.107	0.000 **
Home owning situation	0.084	1.414	0.158
*Variables of the social environment (Level 2)*			
Number of walking trips to total trips	0.193	3.129	0.002 **
*Variables of the built environment (Level 3)*			
Housing type	−0.057	−0.887	0.376
Number of parks and plazas	0.134	2.102	0.036 **
Number of trees per zone	−0.087	−0.975	0.330
Link Node Ratio	−0.134	−1.348	0.179
Intersection Density	0.007	0.085	0.932
Mixed Land Use	0.245	2.612	0.010 **
Access to services	−0.187	−2.032	0.143
Access to educational destinations	−0.122	−1.476	0.141
Access to health canters	0.114	1.450	0.148
Crime rate	0.128	1.541	0.124
Total accident rate	0.109	1.307	0.192
Topography	0.166	2.240	0.026 **
Housing Density	0.048	0.798	0.425

* *p* < 0.05; ** *p* < 0.01. Dependent variable: Walking Behavior; R Square: 0.231.

## Data Availability

Not applicable.
